# Retraction Note: Phytochemistry, antioxidant, anticancer, and acute toxicity of traditional medicinal food Biarum bovei (Kardeh)

**DOI:** 10.1186/s12906-026-05643-5

**Published:** 2026-10-15

**Authors:** Bassam Ali Abed Wahab, Zaenah Zuhair Alamri, Ahmed A.j. Jabbar, Ibrahim Abdel Aziz Ibrahim, Riyad A. Almaimani, Hussain A. Almasmoum, Mazen M. Ghaith, Wesam F. Farrash, Yahya A. Almutawif, Khalid Aidarous Ageeli, Soliman Mohammed Alfaifi, Rahaf Frehan Alharthi

**Affiliations:** 1 Department of Physiology, Biochemistry & Pharmacology, Faculty of Vet Medicine, University of Kufa, Kufa, Iraq; 2 Department of Biology, College of Science, University of Jeddah, Jeddah, Saudi Arabia; 3 Department of Medical Laboratory Technology, Erbil Technical Health and Medical College, Erbil Polytechnic University, Erbil, 44001 Iraq; 4 Department of Pharmacology and Toxicology, Faculty of Medicine, Umm Al-Qura University, Makkah, Saudi Arabia; 5 Department of Biochemistry, Faculty of Medicine, Umm Al-Qura University, Makkah, Saudi Arabia; 6 Department of Laboratory Medicine, Faculty of Applied Medical Sciences, Umm Al-Qura University, Makkah, Saudi Arabia; 7 Department of Medical Laboratories Technology, College of Applied Medical Sciences, Taibah University, Madinah, 42353 Saudi Arabia; 8 Department of Pharmacy, Faculty of Pharmacy, Jazan University, Jizan, Saudi Arabia

**Retraction Note: BMC Complement Med Ther 23, 283 (2023)**

**https://doi.org/10.1186/s12906-023-04080-y**

The Editors have retracted this article. After publication, concerns were raised about some of the data in this Article. Specifically, similarities were detected:

- between all six panels of Fig. 7 in this article and all six panels of Fig. 1 in [1];
- between the upper-right part of Fig. 7E in this article and lower-left part of Fig. 2B (kidney) in [2]; and
- between the left part of Fig. 7B and right part of Fig. 7D in this article.

The Authors have been unable to address these concerns.

The Editors have therefore lost confidence in the data and conclusions of this article.

The Authors did not respond to any correspondence from the Publisher about this retraction.
